# Supplementary material for: Cancer Vaccination against Extracellular Vimentin Efficiently Adjuvanted with Montanide ISA 720/CpG
Source: Cancers (Basel). 2022 May 24;14(11):2593. doi: 10.3390/cancers14112593 (PMC9179438; doi:10.3390/cancers14112593)
Supplement: Supplementary file 1 [file cancers-14-02593-s001.zip › cancers-1652140-supplementary.pdf]

cancers

MDPI

Supplementary Materials

Cancer Vaccination against Extracellular Vimentin Efficiently Adjuvanted with Montanide ISA 720/CpG

Karlijn van Loon, Elisabeth J. M. Huijbers, Jan David de Haan and Arjan W. Griffioen

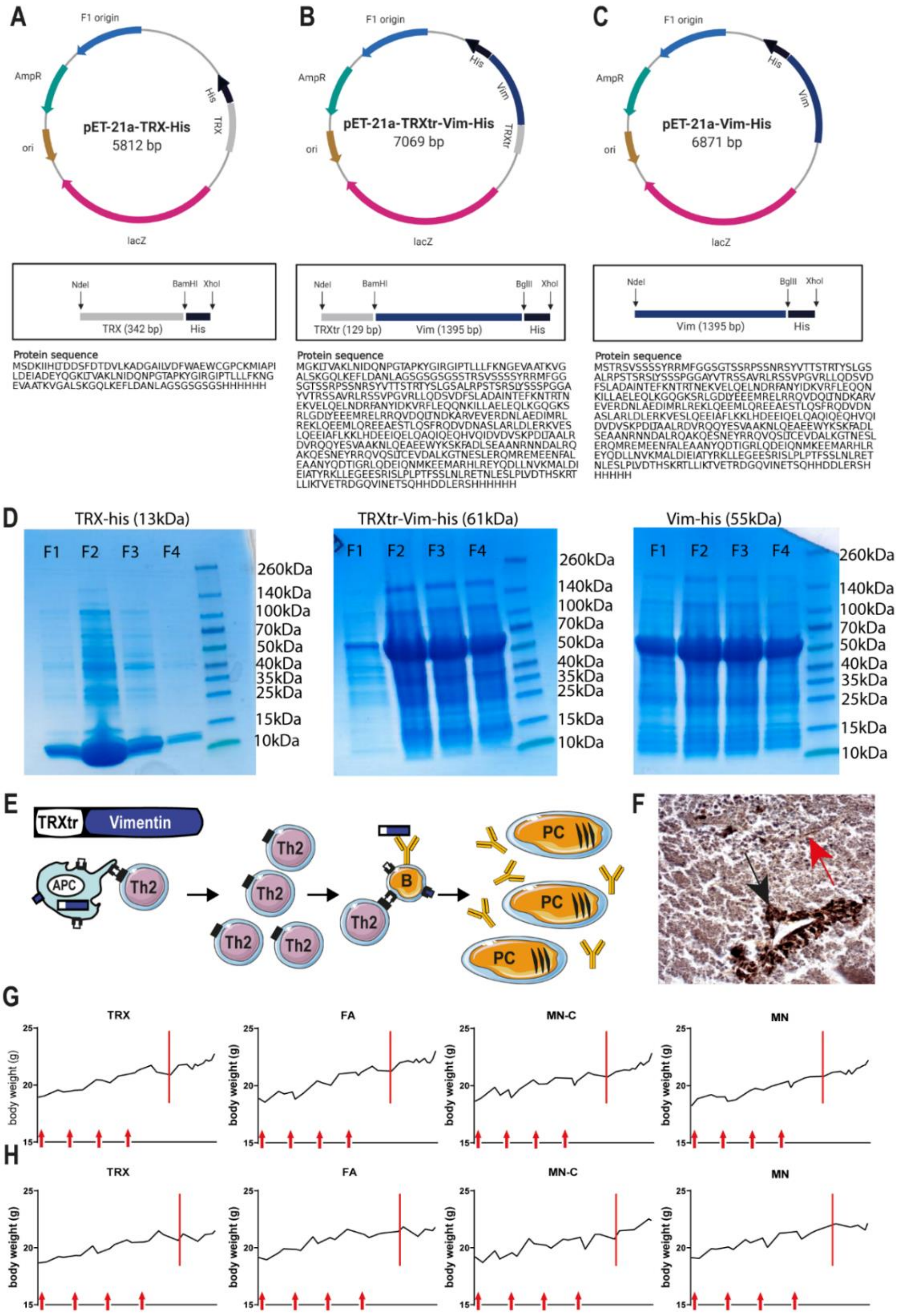

Figure S1. (A-C) The pET21a expression vector for production of the different protein constructs TRX-his (A), TRXtr-Vim-His (B), and Vim-his (C). The DNA sequences of the different protein

constructs (insert) were inserted between the restriction sites Nde1, BamH1, and Xho1 in the multiple cloning site, as indicated in the black rectangle. The full amino acid sequence of the proteins including a polyhistidine-tag (His) is indicated below the vector. The Vim-his protein was used for the detection of anti-vimentin antibodies in ELISA and affinity determination using Biacore. (D) SDS page gel of the purified recombinant proteins. TRX (13kDa) was eluted with four fractions of 200mM imidazole. TRXtr-Vim (61kDa) and Vim (55kDa) wee eluted with four fractions of elution buffer with 8M urea and pH 4.5. (E) The mechanism for breaking self-tolerance using iBoost technology. TRXtr-Vim was internalized by antigen-presenting cells (APC) and presented on MHC class II molecules. TRXtr-specific T helper cells (Th2) will become activated and clonally expand. Vimentin-specific autoreactive B cells also internalize TRXtr-Vim via their B cell receptor and present similar peptides on MHC class II. Previously activated TRXtr-specific Th2 cells will recognize TRXtr in the context of MHC class II and activate the autoreactive B cells. These vimentin-specific B cells will develop into anti-vimentin-producing plasma cells (PC). (F) Representative image of a B16F10 tumor stained for vimentin at a 200x magnification. A large blood vessel with positive vimentin staining in the surrounding matrix is indicated with a black arrow, and another small vessel with a positive vimentin staining is indicated with a red arrow. (G-H) Mean body weight of TRX, FA, MN-C, and MN-vaccinated mice of Study I (G) and Study II (H). Red arrows indicate immunization time points. The red vertical line indicates the moment of B16F10 tumor cell inoculation. Figure created with BioRender.com.

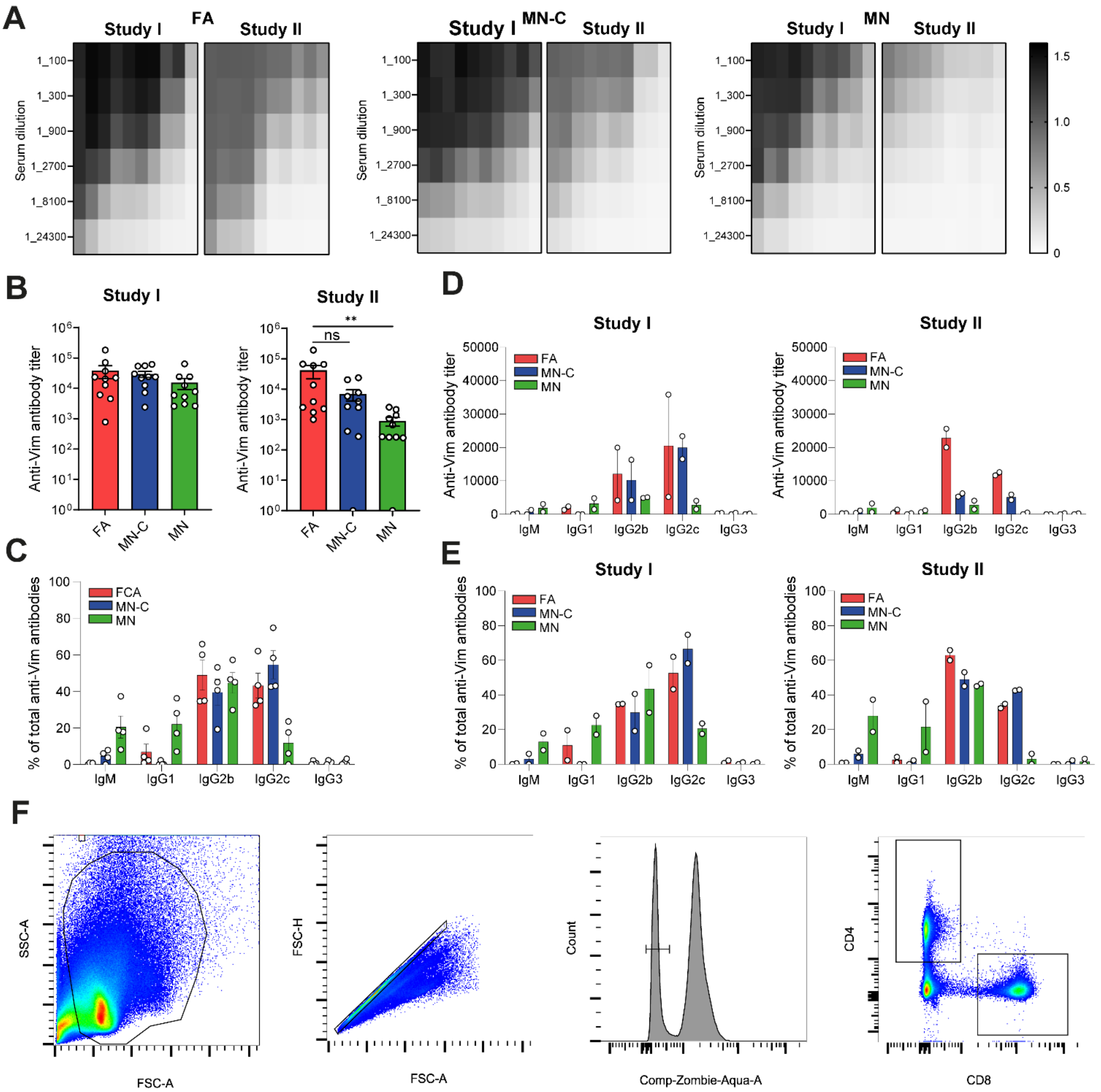

Figure S2. (A) Anti-vimentin total immunoglobulin (Ig) titers for each vaccine group (n = 20) in serum after the fourth vaccination, assessed by ELISA. Each column indicates a different mouse, and the rows represent a serial dilution of the mouse serum. Values presented are optical density measured at 655nm. (B) Anti-vimentin total immunoglobulin (Ig) titers for each vaccine group in serum after the fourth vaccination using ELISA. Antibody titers were calculated based on the intersection between the titration curve and the threshold of OD655 of 0.2. (C) Analysis of vimentin-specific IgG subclasses and IgM after four vaccinations using ELISA, as presented as percentage of total anti-vimentin antibodies. To calculate the percentage of total anti-vimentin antibodies, titers of all five isotypes were summed, and the titer per isotype was divided by the summed titer and multiplied by 100. Graphs contain data from Study I and Study II combined, with sera of 5 mice pooled per dot. (D) Analysis of vimentin-specific IgG subclasses and IgM after four vaccinations using ELISA, with sera of 5 mice pooled per dot. (E) Analysis of vimentin-specific IgG subclasses and IgM after four vaccinations using ELISA, presented as percentage of total anti-vimentin antibodies. Bars represent mean + SEM. (F) Representative gating strategy of splenocytes after 48h of stimulation with recombinant vimentin protein. ** $p < 0.01$.

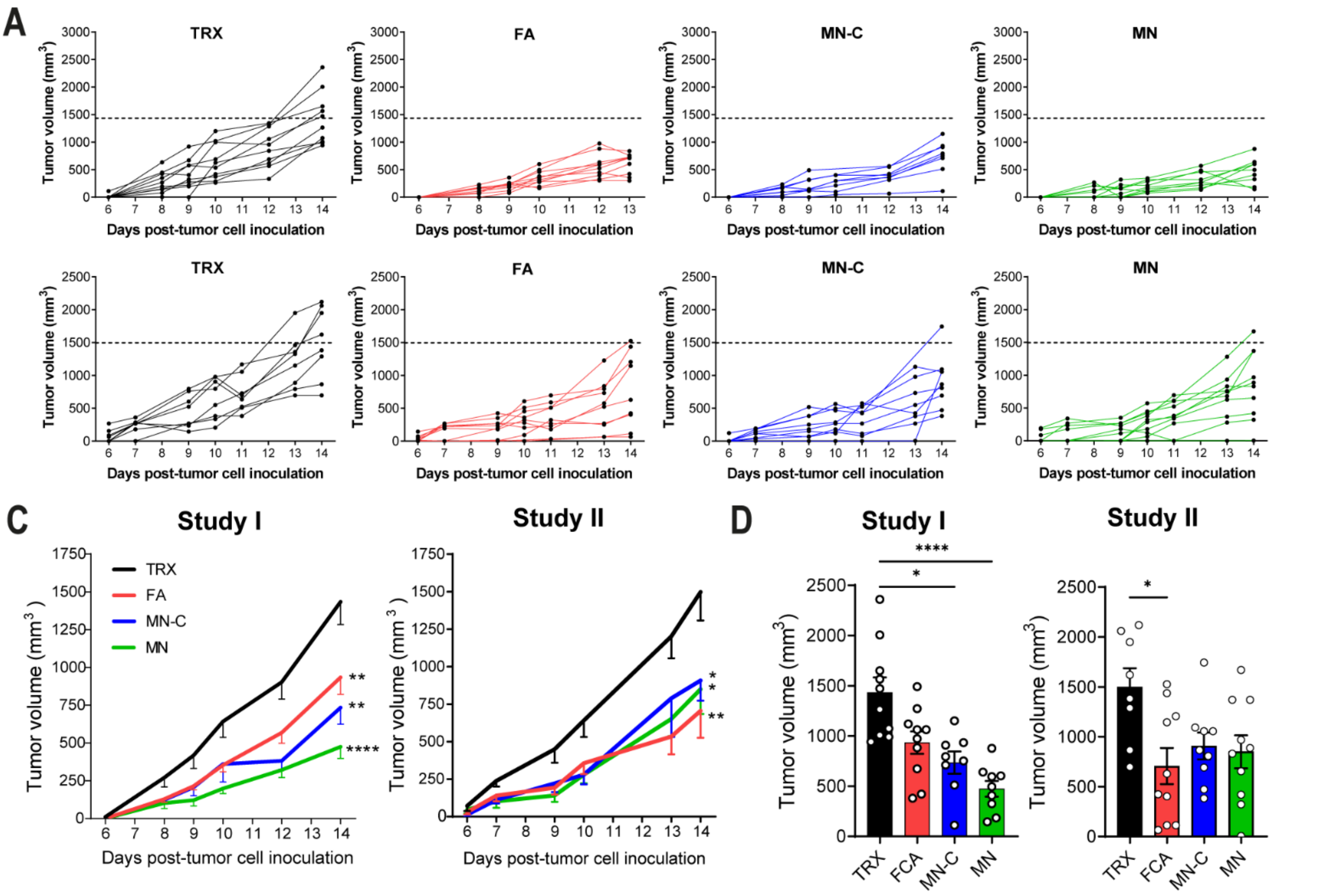

Figure S3. (A-B). Tumor growth curves of each mouse of Study I (A) and Study II (B) up to 14 days post-tumor cell inoculation. The dotted horizontal line indicates the mean tumor volume of the TRX control group at day 14. (C) Tumor growth curves of B16F10 melanoma in vaccinated mice of Studies I and II plotted separately per study. Tumor growth curves are presented as mean + SEM and compared by two-way ANOVA. (D) Final tumor volume 14 days after tumor cell inoculation of Study I (left) and Study II (right) Bars represent mean + SEM.
